# Differentiating Mild Cognitive Impairment, Alzheimer's Disease, and Dementia With Lewy Bodies Using Cingulate Island Sign on Perfusion IMP-SPECT

**DOI:** 10.3389/fneur.2020.568438

**Published:** 2020-11-19

**Authors:** Hidekazu Kanetaka, Soichiro Shimizu, Yuta Inagawa, Daisuke Hirose, Naoto Takenoshita, Hirofumi Sakurai, Haruo Hanyu

**Affiliations:** Department of Geriatric Medicine, Tokyo Medical University, Tokyo, Japan

**Keywords:** cingulate island sign, IMP-SPECT, CIS, Alzheimer's disease, dementia with Lewy bodies (DLB)

## Abstract

The cingulate island sign (CIS) on fludeoxyglucose (FDG)-positron emission tomography (PET) is a supporting biomarker of dementia with Lewy bodies (DLB). Its diagnostic accuracy has only been investigated in FDG-PET, however. The present prospective study compared the CIS on I-iodoamphetamine-single photon emission computed tomography (SPECT) among patients with mild cognitive impairment (MCI), AD, or DLB. Fifty-eight patients with MCI, 42 with probable AD, and 58 with probable DLB were enrolled. The “CIScore” used to evaluate the CIS was defined as the ratio of volume of interest (VOI)-1 (indicating posterior cingulate gyrus [PCG]) to VOI-2 (area of significantly reduced regional cerebral blood perfusion [rCBF] in DLB patients compared with in healthy controls). It was calculated using eZIS software. The CIScore for MCI, DLB, and AD was 0.22, 0.23, and 0.28, respectively. The CIScore in the AD group was significantly higher than that in the DLB or MCI groups (AD vs. DLB: *p* < 0.001, AD vs. MCI: *p* < 0.005). This suggests that the CIScore can discriminate DLB from AD, if the decrease in rCBF in the PCG is similar between them. We believe that it is difficult to identify MCI based on the CIScore, as the decrease in rCBF in the PCG is not severe. The diagnostic accuracy of the CIScore may be low as it often shows an increase in elderly DLB patients, in whom the pathologically common form is most prevalent (1). Further study should include assessment of multiple components such as symptom classification and age.

## Introduction

It is clinically important to distinguish dementia with Lewy bodies (DLB) from Alzheimer's disease (AD) as the type of treatment required for each differs. There are a number of characteristics particular to patients with DLB. These include occipital hypoperfusion, and greater preservation of metabolism in the posterior cingulate gyrus (PCG) than in the precunei, the latter being known as the cingulate island sign (CIS).

The CIS on FDG-PET is recognized as a supporting biomarker of DLB according to the International DLB Diagnostic Criteria published in 2017 (2, 3). Although the diagnostic accuracy of the CIS has been investigated in FDG-PET (4, 5), to our knowledge, it has yet to be determined in brain perfusion SPECT.

One previous study (6) investigated the diagnostic accuracy of the CIS on technetium-99m ethyl cysteinate dimer (ECD)-SPECT in a cohort of 13 patients with DLB and 13 with AD. The results revealed DLB-specific VOIs in areas showing a significant decrease in brain perfusion in the DLB group. As numerators of these ratios, early Alzheimer's disease-specific VOIs were used after subtracting DLB-specific VOIs. The DLB-specific VOIs were used as the denominator. The CIScore was generated from the SPECT Z-scores using the Z-score imaging system (eZIS). The results indicated that DLB could be differentiated from AD by this method with an accuracy, sensitivity, and specificity of 84.6, 92.3, and 76.9%, respectively, with a threshold value of 0.281 (CIScore).

One study reported that ^123^-I-iodoamphetamine (IMP)-SPECT faithfully reflected cerebral blood flow, even in areas where it was high, whereas ECD showed a weaker correlation (7). This suggests that IMP is more likely to capture mild change in blood flow.

It should also be noted that IMP is the more suitable option in observation of the cerebral cortex, as cerebellar accumulation is less than that in the cerebral cortex. This modality offers the best linearity with true cerebral blood flow among the three formulations of SPECT available. Therefore, we believe that IMP offers the best means of capturing mild change in cerebral blood flow.

The purpose of this study was to compare the CIS on IMP-SPECT among patients with mild cognitive impairment (MCI), AD, or DLB.

## Methods

### Ethics

Written informed consent was obtained from the patients or their next of kin/legal guardian if they were a minor for the publication of any potentially identifiable images or data included in this article.

The study protocol was approved by the Ethics Committee of Tokyo Medical University (Approval no: 3304).

### Patients

The participants in this prospective study comprised patients with MCI, AD, or DLB attending the Memory Clinic at our hospital. All MCI patients were categorized as the amnestic type. The exclusion criteria were any medication such as benzodiazepine, antidepressants, or antipsychotics.

A total of 58 patients with MCI, 42 with probable AD, and 58 with probable DLB were enrolled (Table 1).

**Table 1:** Patient characteristics.

	**MCI**	**AD**	**DLB**
Total: 158	58	42	58
Age (average)	54–97 (77.5)	52–91 (78.8)	70–94 (81.5)^#^^†^
MMSE	26.6 ± 1.9	20.9 ± 2.9^**^	20.5 ± 5.0^**^
MoCA-J	20.1 ± 3.0	17.5 ± 4.3	14.3 ± 4.6^*^
ADAS-Jcog	8.8 ± 3.5	16.4 ± 4.8^*^	16.5 ± 7.3^*^
WMS-R (logical memory I)	11.5 ± 6.4	5.9 ± 3.4^**^	7.3 ± 4.8
	6.6 ± 6.1	4.0 ± 3.7	3.1 ± 4.4

# *p <0.05*,

* *p <0.01*,

** p <0.001, vs. MCI

† *p <0.01, vs. AD*.

*MMSE, Mini-Mental State Examination; MoCA-J, Japanese version of Montreal Cognitive Assessment; ADAS-Jcog, Alzheimer's Disease Assessment Scale-cognitive component-Japanese version; WMS-R, Wechsler Memory Scale-Revised*.

The diagnosis of MCI or AD was based on the National Institute on Aging and Alzheimer's Association criteria for a diagnosis of MCI or probable AD (8). The diagnosis of DLB was based on the updated diagnostic criteria published in 2017 by the International DLB Consortium.

None of the patients with MCI or probable AD showed fluctuating cognition, visual hallucinations, parkinsonism, or REM sleep behavior disorder (RBD) as determined by geriatric neurologists.

Some of the patients with probable DLB showed fluctuating cognition (22%), visual hallucinations (53%), parkinsonism (45%); Hoehn & Yahr stage 2.3 ± 1.3, or RBD (40%). None of the patients were taking any medication such as benzodiazepine, antidepressants, or antipsychotics.

The mean ages of the patients in the MCI, AD, and DLB groups were 77.5 ± 8.8, 78.8 ± 7.1, and 81.5 ± 5.5 years, respectively. Although no significant difference was observed in age between the MCI and AD groups, it was significantly higher in the DLB group. All the patients underwent a general physical examination. They were also required to undergo the following neurological and neurocognitive tests: Mini-Mental State Examination, MMSE (9); the Japanese version of the Montreal Cognitive Assessment, MoCA-J; the Japanese version of the Alzheimer's Disease Assessment Scale-cognitive component, ADAS-Jcog (10); and the Wechsler Memory Scale-Revised logical memory 1, WMS-R (11). All the patients were also required to undergo IMP-SPECT.

SPECT imaging was performed using a triple-head rotating gamma camera (PRISM 3000 XP, Picker). Imaging was commenced at 15 min following intravenous administration of 222 MBq of N-isopropyl-p-[123I] iodoamphetamine. The slice thickness was 4.3 mm (128 × 128 matrix).

### Regarding the Z-Score, eZIS System, and CIScore

The details of the eZIS have been described previously (12, 13). Briefly, voxel-by-voxel Z-score analysis was performed after voxel normalization to global mean or cerebellar values. The Z-score was derived according to the following formula: ([healthy control mean]—[individual patient value])/(healthy control SD).

A database of values obtained from healthy controls was then constructed by Matsuda et al. (12) at the National Center of Neurology and Psychiatry, which included data on 40 cognitively healthy individuals aged between 70 and 87 years.

The eZIS is considered to be very useful in diagnosing dementia in routine studies, because the degree of expertise of the observer does not influence the result, and a common database is available for converting site-specific SPECT data to core data.

**Figure 1:**
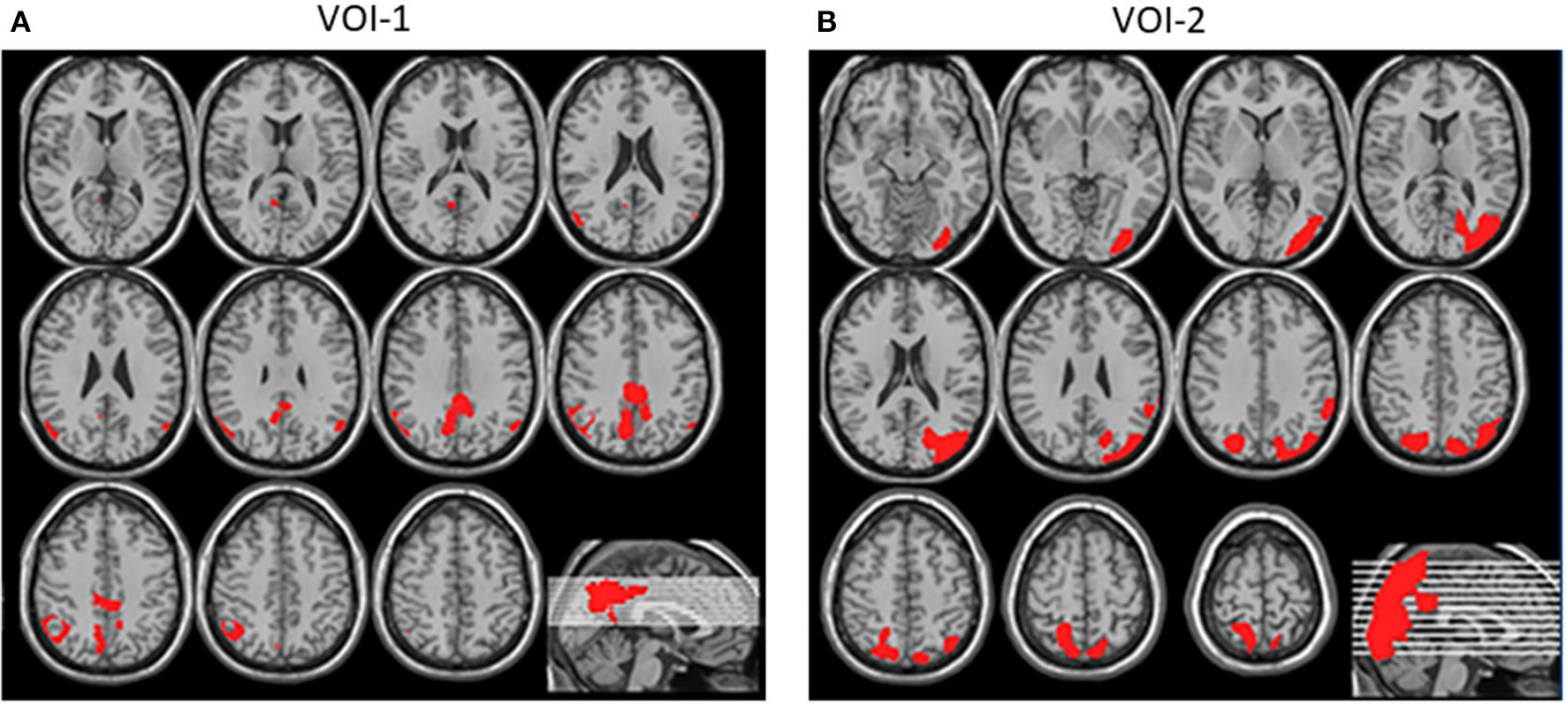
**(A)** VOI-1, shown in red, was defined as indicating PCG, an area characteristically involved in AD. **(B)** VOI-2, shown in red, was defined as area indicating significant reduction in cerebral perfusion in DLB patients compared with in healthy controls (Quotes from reference 5).

This program also allows statistical parametric mapping results to be incorporated into an automated analysis of the Z-score values in the volume of interest (VOI). The new version of the eZIS program has the following two types of VOI: VOI-1, which indicates the PCG, an area characteristically involved in AD; and VOI-2, an area defined as indicating a significant reduction in cerebral perfusion in DLB patients compared with in healthy controls (Figure 1). The CIScore is defined as the ratio of the VOI-2 to the VOI-1 Z-score, and is automatically calculated by the eZIS software, as is the Z-score for each VOI. The CIS is only a visual assessment, while the CIScore is a quantitative parameter.

### Statistical Analysis

Values are expressed as the mean ± SD. Comparisons among the three groups were performed with a one-way analysis of variance (ANOVA); a *post-hoc* Tukey-Kramer test was used to analyze changes in the multidimensional rating scales in each group.

A *p*-value of < 0.05 was considered to indicate a statistically significant difference between two groups. All the analyses were performed with “IBM SPSS Statistics (Chicago, IL).”

The discrimination rate of the CIScore between AD and DLB was determined using ROC analysis. The cutoff value was defined by the Youden index.

## Results

The results of the neurological and neurocognitive tests were as follows: the MMSE scores in the MCI, AD, and DLB groups were 26.6 ± 1.9, 20.9 ± 2.9, and 20.5 ± 5.0; the MoCA-J scores 20.1 ± 3.0, 17.5 ± 4.3, and 14.3 ± 4.6; the ADAS-Jcog scores 8.8 ± 3.5, 16.4 ± 4.8, and 16.5 ± 7.3; and the WMS-R scores 6.6 ± 6.1, 4.0 ± 3.7, and 3.1 ± 4.4, respectively.

**Figure 2:**
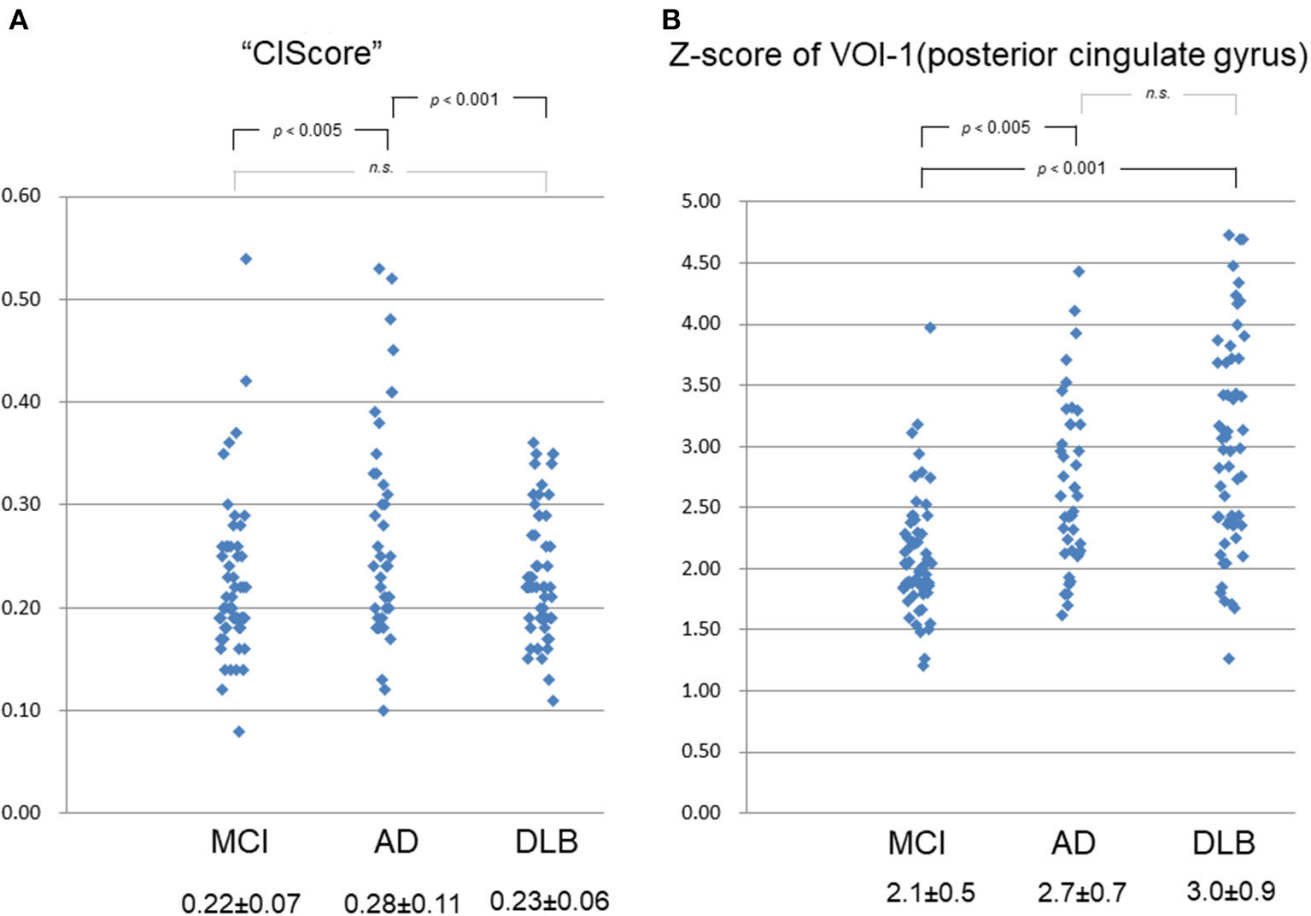
**(A)** CIScore in MCI, AD, and DLB groups was 0.22 ± 0.07, 0.28 ± 0.11, and 0.23 ± 0.06, respectively. CIScore in AD group was significantly higher than that in DLB or MCI group (AD vs. DLB: *p* < 0.001, AD vs. MCI: *p* < 0.005). No significant difference was observed between MCI and DLB groups, however. **(B)** Z-scores of VOI-1 in MCI, AD, and DLB groups were 2.1 ± 0.5, 2.7 ± 0.7, and 3.0 ± 0.9, respectively. Scores in AD and DLB groups were significantly higher than that in MCI group (AD vs. MCI: *p* < 0.005, DLB vs. MCI: *p* < 0.001). No significant difference was observed between scores in AD and DLB groups, however.

The ANOVA revealed a significant difference in the CIScore among the three groups (*p* < 0.001). The CIScore in the MCI, AD, and DLB groups was 0.22 ± 0.07, 0.28 ± 0.11, and 0.23 ± 0.06, respectively (Figure 2A). The CIScore in the AD group was significantly higher than that in the DLB or MCI groups (AD vs. DLB: *p* < 0.001, AD vs. MCI: *p* < 0.005). Receiver operating characteristic analysis revealed a significant difference in the CIScore between the AD and DLB groups. The cut-off value of the CIScore was determined to 0.23 according to the ROC analysis (Youden index). The area under the curve was 0.620 (*p* < 0.05); and sensitivity and specificity were 0.50 and 0.73, respectively. No significant difference was observed between the MCI and DLB groups, however.

The Z-scores in the MCI, AD, and DLB groups were 2.1 ± 0.5, 2.7 ± 0.7, and 3.0 ± 0.9, respectively (Figure 2B). A significant difference was observed in the Z-scores for VOI-1 (which demonstrated a decrease in blood perfusion flow in the PCG) between the MCI group and each other group (*p* < 0.001 by ANOVA). The scores in the AD and DLB groups were significantly higher than that in the MCI group (AD vs. MCI: *p* < 0.005, DLB vs. MCI: *p* < 0.001). No significant difference was observed between the scores in the AD and DLB groups, however.

## Discussion

**Figure 3:**
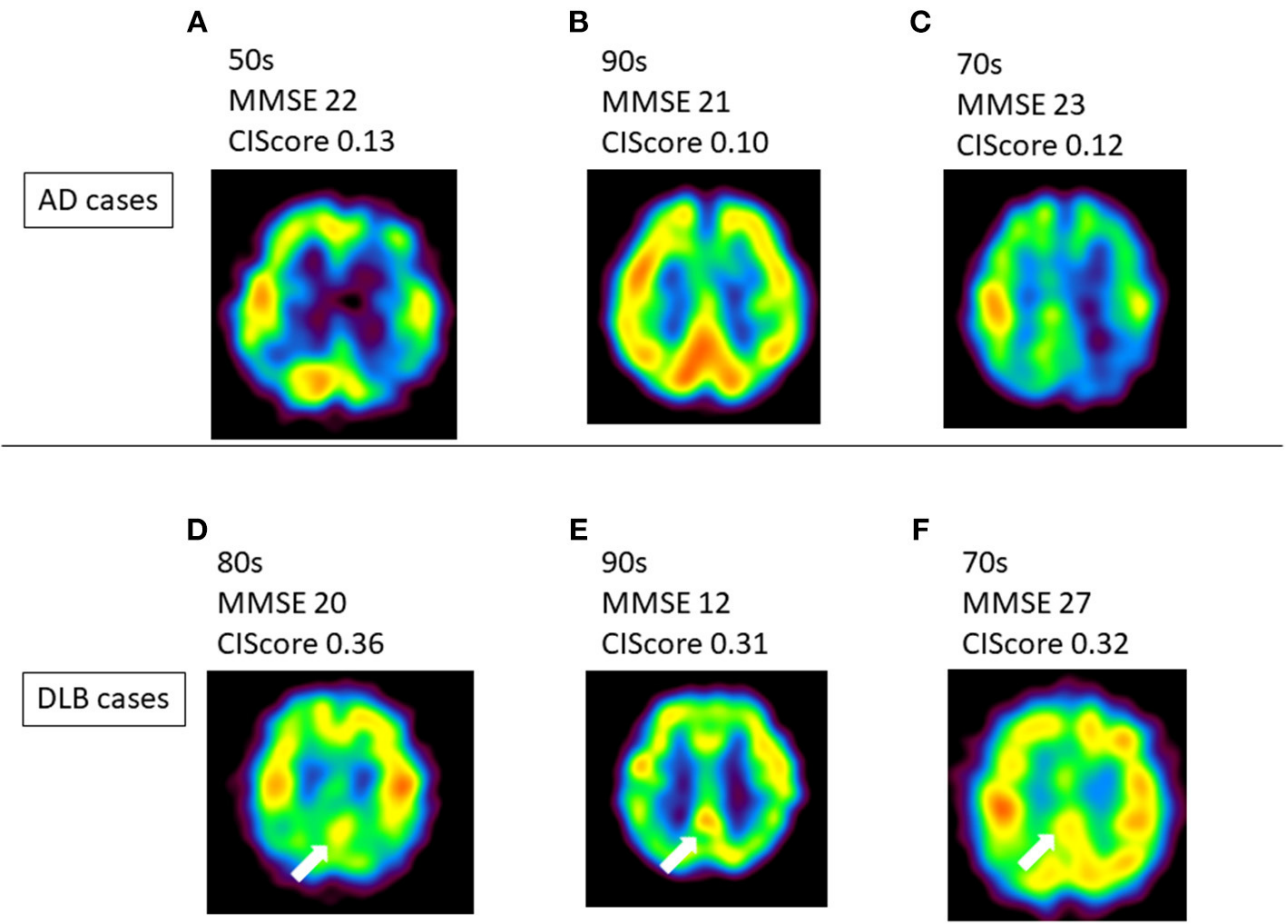
**(A–C)** Several Alzheimer's disease (AD) patients showed no CIS. **(D–F)** Typical dementia with Lewy bodies (DLB) cases showed CIS on IMP-SPECT. CIS is indicated by white arrow in these images. There were some cases that it is hard to detect CIS like case-(f). CIS, Cingulate Island sign; MMSE, Mini-Mental State Examination.

The present results suggest that the CIScore can be used to differentiate DLB from AD when patients show similar levels of decrease in regional cerebral blood flow (rCBF) in the PCG. In Japan, SPECT is widely used in the diagnosis of dementia. Although a diagnosis may be possible by gross examination in some typical cases (Figure 3), a qualitative diagnosis offers the potential of improved efficiency in this respect. Analysis of the CIScore may increase the value and usefulness of IMP-SPECT as an inexpensive and simple method of differentiating DLB from AD (14, 15).

In the present study, the CIScore in the MCI patients was low, making it difficult to detect. This was because the decrease in rCBF in the PCG was not severe. However, as DLB patients may be affected by the coexistence of AD pathologies, there is a significant difference in the PCG between MCI and DLB.

As rCBF in VOI-1 (PCG) in the MCI patients was maintained, no significant difference was observed in the CIScore. This suggests that it is difficult to detect DLB when it is at the MCI stage. Therefore, observing the progress of dementia may be required to avoid misdiagnosis. In addition, the diagnostic accuracy of the CIScore may be low. The diagnostic accuracy of the CIScore may be low as it often shows an increase in elderly DLB patients, in whom the pathologically common form is most prevalent (1). Further study should include assessment of multiple components such as symptom classification and age.

The ability to cross-reference different databases using the eZIS system may greatly enhance the diagnostic value of brain SPECT imaging, however. It is possible that defining an IMP-SPECT-specific VOI would improve the identification rate between MCI, AD, and DLB, although further study is required to confirm this.

Future studies assessing multiple components (such as symptom classification, age, etc.) are required to determine the accuracy of the present method. The presence or absence of hallucinations, in particular, may be affected by occipital lobe blood flow. Determining the clinical characteristics in DLB patients with low CIScores may lead to greater accuracy in diagnosis.

This study had several crucial limitations. Firstly, the diagnostic utility of the CIScore in discriminating DLB from AD may not be high due to the coexistence of DLB and AD pathologies. Here, the diagnosis of AD or DLB was based on diagnostic criteria (those of the National Institute of Neurological and Communicative Disorders and Stroke and Alzheimer's Disease and Related Disorders Association, and the DLB International Workshop), and complications could not be completely ruled out. The DLB International Workshop criteria revealed the coexistence of DLB and AD pathologies. Most patients with DLB also demonstrate AD pathology, including cortical amyloid plaques and neurofibrillary tangles. Therefore, we hypothesized that the absence of the CIS in DLB patients would suggest the coexistence of DLB and AD pathologies, whereas its presence would indicate DLB alone. Our next step in future study, therefore, will be to investigate the CIScore in patients in whom DLB is confirmed pathologically.

Secondly, the absence of healthy controls in the present study is not a trivial limitation. However, fundamental ethical problems associated with the use of imaging techniques in healthy controls in the field of nuclear medicine make this problematic.

Thirdly, this study was carried out at a single memory disorder clinic; therefore, the number of patients enrolled was relatively small, and the age of the patients in the DLB group was significantly higher than that in the AD or MCI groups.

Fourthly, only clinical interviews were used to assess the DLB patients. Further study should also incorporate objective measurements such as the Cognitive Fluctuation Inventory and the Unified Parkinson's Disease Rating Scale.

Further study should also include additional amyloid PET. Such studies should also be multi-center and include larger patient cohorts, including a healthy control group. The results of pathological examination will also be required to confirm the validity of the results.

In conclusion, the present results showed a significant difference among the three groups using IMP. This suggests that the diagnostic power of IMP is not inferior to that of ECD. They also suggest that the CIScore in brain perfusion IMP-SPECT is useful in clinically differentiating DLB from AD.

## Data Availability Statement

The original contributions presented in the study are included in the article/supplementary materials, further inquiries can be directed to the corresponding author/s.

## Ethics Statement

The studies involving human participants were reviewed and approved by MAMORY SUZUKI, Ethics Committee of Tokyo Medical University (Approval no: 3304). The patients/participants provided their written informed consent to participate in this study. Written informed consent was obtained from the patients or their next of kin/legal guardian if they were a minor for the publication of any potentially identifiable images or data included in this article.

## Author Contributions

SS, HS, and HH: patients recruitment. YI, DH, and NT: data assessment. All authors contributed to the article and approved the submitted version.

## Conflict of Interest

The authors declare that the research was conducted in the absence of any commercial or financial relationships that could be construed as a potential conflict of interest.
